# Mode of Transport and Patient Status Upon Arrival at a Tertiary Pediatric Emergency Department in a Developing Country: A Prospective Observational Study

**DOI:** 10.7759/cureus.68067

**Published:** 2024-08-28

**Authors:** Jency Antony, Narayanan Parameswaran, Ramanathan R, Rajasekaran K, Sriram Pothapregada, Senthil Kumar

**Affiliations:** 1 Pediatrics, Jawaharlal Institute of Postgraduate Medical Education & Research, Puducherry, IND; 2 Pediatrics and Child Health, Jawaharlal Institute of Postgraduate Medical Education & Research, Puducherry, IND; 3 Pediatrics, Rajah Muthiah Medical College, Chidambaram, IND; 4 Pediatrics, Government Tiruvannamalai Medical College and Hospital, Tiruvannamalai, IND; 5 Pediatrics and Neonatology, All India Institute of Medical Sciences, Bilaspur, Bilaspur, IND; 6 Pediatrics, Government District Headquarters Hospital, Cuddalore, IND

**Keywords:** status at arrival, triage, pediatric emergency, pre-hospital transport, ambulance

## Abstract

Introduction

In children with acute illness or injury, timely access to healthcare is crucial for optimal outcomes, including early identification, prompt stabilization, and appropriate referral. Prehospital transport practices often represent a weak link in the system and are frequently underreported. Enhancing these practices could greatly benefit pediatric care. This study aims to analyze the mode of transport and condition upon arrival of children at our hospital’s pediatric emergency department.

Methods

This hospital-based prospective observational study was conducted from August 2019 to March 2020. Data on referral status, mode of transport, vital parameters at admission, and final outcomes in terms of survival were collected prospectively from patient admission records.

Results

Out of 1,658 children, 1,326 (80%) were referred from another health facility. Among these, only 10.8% received prior notification about their arrival. The most common mode of transport was a bus (39.3%), followed by locally available ambulances (24.4%), two-wheelers (15.6%), and cars (10.9%). In terms of medical conditions, 47.5% of the children had respiratory infections, 15.8% had renal ailments, and 14.1% had neurological complaints. Upon arrival, 1,214 (73.2%) were in stable condition, while 444 (26.8%) were unstable. Common alterations in vital parameters included hypothermia (16.6%), SpO2 <92% (9.9%), abnormal heart rate (32.5%), and abnormal respiratory rate (56%). Additionally, 111 (6.6%) children required CPR upon arrival.

Conclusions

The data highlights the need for improvements in our referral systems and the modes of transport for sick children within our population.

## Introduction

Pediatric healthcare networks serve millions of children annually, addressing both acute illnesses and injuries that profoundly impact children and their families emotionally [1]. Timely and efficient access to hospital care is crucial for their health and well-being. However, inadequate transportation remains a significant barrier to effective healthcare delivery. Efficient triage, resuscitation, and stabilization in pediatric emergencies are contingent on the timely transport of children to healthcare facilities [1].

The Integrated Management of Childhood Illness strategy, endorsed by WHO and UNICEF, aims to enhance child health through community and household care, primary care, good referral practices, and care at first-level hospitals. According to current guidelines, approximately 10-20% of severely ill children presenting for primary care require initial treatment or stabilization at a first referral or district hospital [2].

Existing referral systems from peripheral hospitals to tertiary care centers face several challenges. Referral and transport have been identified as the weakest links in the healthcare system [3]. Prehospital transport practices for sick children are often underreported [3]. Common issues include a lack of pre-transport communication, poor documentation, and inadequate transport services to meet resuscitation needs [4]. Many children arrive via public transport, which may be due to a lack of awareness about available free patient transport services, financial constraints, or referral from remote areas where public transport is the only option [5].

Improving the referral system could significantly enhance the health and outcomes of millions of children each year. Our study seeks to address knowledge gaps regarding the current state of referral systems for children attending our hospital.

## Materials and methods

We conducted a hospital-based prospective observational study over eight months, from August 2019 to March 2020, at a tertiary care hospital in South India. Upon arrival at the pediatric emergency department, all children were assessed at the triage desk by pediatric resident doctors and classified into triage Levels 1 through 5, with Level 1 indicating the most critical condition and Level 5 the least urgent. Level 1 represented immediate, life-threatening situations, while Level 2 included emergencies that could potentially become life-threatening. Levels 3 and 4 were categorized as urgent and semi-urgent, respectively, indicating non-life-threatening conditions, and Level 5 referred to non-urgent cases that required treatment when time permitted.

Children classified as Levels 1 and 2 were considered “unstable,” while those categorized as Levels 3, 4, and 5 were deemed “stable.” Children in Levels 3 and 4 were initially managed in the emergency ward and later transferred to either a routine pediatric ward or the pediatric intensive care unit (PICU) based on their condition and response to treatment. Level 5 children were discharged from the emergency room after an appropriate observation period.

Data collected for the study included baseline characteristics such as age, sex, date and time of presentation, referral status, mode of transport, chief complaints, vital signs at presentation, and triage level. This information was recorded in the triage register. Outcome data, including transfers to the pediatric ward or PICU, discharge, death, or leaving against medical advice, were obtained from the admission register and the hospital information system. For children admitted to the pediatric ward and PICU, the duration of admission and final outcome at discharge, including survival or death, were also recorded.

Sample size

This prospective observational cohort study was conducted over eight months, from August 2019 to March 2020. It included all children aged between 30 days and 12 years who were admitted to our pediatric emergency department. On average, six to eight children meeting these criteria were admitted daily, leading to a convenient sample size of 1,600 for the study.

Statistical analysis

Data were collected using a structured proforma and analyzed with IBM SPSS Statistics for Windows, Version 20.0 (Released 2011; IBM Corp., Armonk, NY, USA). Continuous variables, such as age, weight, height, and duration of illness, were summarized as mean (SD) or median (IQR). Categorical variables, including gender, diagnosis at admission, final diagnosis, type of referring hospital, mode of transport, and whether the primary care physician contacted our hospital before referring, were summarized as proportions. Survival rates and duration of hospital stay were expressed as percentages. Vital parameters and mode of transport were compared using chi-square analysis for categorical variables, or Fisher’s exact test when appropriate.

Ethical consideration

The study protocol was approved by the Institute Ethics Committee (IEC) prior to the commencement of the study (approval number JIP/IEC/2018/0200).

## Results

During the study period, a total of 1,658 pediatric patients were admitted to the emergency department. Among these, 747 patients (45%) were aged one to 12 months, with a median age of 44 months (IQR: 8-84). The majority of patients were boys (1,016, 61.3%). Most patients (1,326, 80%) arrived without prior contact between the referring health facility and our emergency department. Respiratory complaints were noted in 477 patients (28.7%), neurological complaints in 271 (16.3%), and renal complaints in 168 (10.1%). The predominant modes of transport were private vehicles: buses (654, 39.3%), cars (341, 20.8%), and motorcycles (259, 15.6%), followed by locally available ambulances (404, 24.3%) (Table 1).

**Table 1:** Clinico-epidemiological characteristics of study children (N = 1,658) ^*^ “Other vehicles” includes buses, two-wheelers, and four-wheelers.

Category	Total (N = 1,658), n (%)
Age	
1-12 months	747 (45)
1-2 years	163 (9.8)
3-5 years	270 (16.3)
6-9 years	275 (16.5)
10-12 years	203 (12.2)
Gender	
Male	1,016 (61.3)
Female	642 (38.7)
Mode of transport	
Ambulance	404 (24.3)
Other vehicles*	1,254 (75.6)
Source of patients	
Referred from another facility (n = 1,326 (80%))	
Medical college	754 (45.4)
Government hospital	402 (24.2)
Private clinic	170 (10.2)
Directly arrived at the emergency department	332 (20)
Contact before transport (N = 1,326)	
Not contacted	1,183 (89.2)
Contacted	143 (10.8)
Intubated before referring (N = 1,326)	
Not intubated	1,208 (91.1)
Intubated	118 (8.9)
CPR (N = 1,658)	
CPR not given in emergency	1,547 (93.3)
CPR given in emergency	111 (6.6)
Secure IV access available (N = 1,326)	
No IV line	1,064 (80.2)
IV line	262 (19.8)
Triage status (N = 1,658)	
Level 1	144 (8.6)
Level 2	300 (18)
Level 3	936 (56.5)
Level 4	272 (16.4)
Level 5	6 (0.4)
Outcome (N = 1,658)	
Expired	222 (13.4)
Survived	1,436 (86.6)

At arrival, 1,214 patients (73.2%) were in stable condition, while 444 patients (26.8%) were unstable (Table 1). Although many patients were referred from medical colleges (754, 45.4%) and government hospitals (402, 24.2%), prior contact before referral was noted in only 10.8% of cases.

Among those transported, hypothermia was more common in patients transported by private vehicles (224, 17.8%) compared to those transported by ambulances (52, 12.8%). However, low oxygen saturation was more frequent among those transported by ambulances (60, 14.8%) compared to those transported by private vehicles (105, 8.4%), with the difference being statistically significant. The rates of tachycardia and tachypnea were similar between the two transport groups. Mortality was higher among patients transported by ambulances (104, 25.7%) compared to those transported by private vehicles (118, 9.4%) (Table 2). Additionally, a higher proportion of patients arriving by ambulance had IV-line access (172, 43.9%) compared to those arriving by private vehicles (90, 9.6%) (Table 3).

**Table 2:** Comparison of vital parameters and outcomes by mode of transport in study participants (N = 1,658*) ^*^ Includes children who arrived directly at the emergency department (N = 332) and those referred from another health facility (N = 1,326). ^†^ All parameters are represented as n (%). ^‡^ “Other vehicles” includes buses, two-wheelers, and four-wheelers.

Vital parameters^†^	Mode of transport		Total (N = 1,658)	p-value	Chi-square value
	Ambulance (n = 404)	Other vehicles^‡^ (n = 1,254)			
Temperature				0.021	5.4868
Hypothermia	52 (12.8)	224 (17.8)	276 (16.6)		
Normothermic/febrile	352 (87)	1,030 (82)	1,382 (83.3)		
SpO₂				0.001	14.31
<92	60 (14.8)	105 (8.4)	165 (9.9)		
≥92	344 (85)	1,149 (91.6)	1,493 (90)		
Heart rate				0.12	4.1638
Normal	256 (63.4)	862 (68.7)	1,118 (67.4)		
Bradycardia	27 (6.6)	77 (6.1)	104 (6.2)		
Tachycardia	121 (29.9)	315 (25)	436 (26.3)		
Respiratory rate				0.092	4.9177
↑RR	193 (47.7)	625 (49.8)	818 (49.3)		
↓RR	37 (9.1)	75 (5.9)	112 (6.7)		
Normal	174 (43)	554 (44)	728 (43.9)		
CPR				0.001	175.9594
Yes	85 (21)	26 (2)	111 (6.7)		
No	319 (78.9)	1,228 (97.9)	1,547 (93.3)		
Outcome				0.001	70.2864
Expired	104 (25.7)	118 (9.4)	222 (13.3)		
Survived	300 (74.3)	1,136 (90.5)	1,436 (86.6)		

**Table 3:** Characteristics of children referred from another facility (N = 1,326*) ^*^ From the total study population (N = 1,658), this table includes only those referred from another health facility, excluding children who arrived directly from home (N = 332). ^†^ “Other vehicles” includes buses, two-wheelers, and four-wheelers.

Vital parameters	Mode of transport		Total (N = 1,326), n (%)	p-value	Chi-square value
	Ambulance (n = 391)	Other vehicles^†^ (n = 935)			
Contacted before referring				0.001	89.8989
Yes	91 (23.5%)	52 (5.5%)	143 (10.7)		
No	300 (76.5%)	883 (94.4%)	1,183 (89.2)		
Intubation before referring				0.001	167.598
Yes	96 (24.5%)	22 (2.4%)	118 (8.8)		
No	295 (75.4%)	913 (97.6%)	1,208 (91.1)		
Secure IV line access available				0.001	205.352
Yes	172 (43.9%)	90 (9.6%)	262 (19.7)		
No	219 (56%)	845 (90.4%)	1,064 (80.2)		

When examining the association between survival and mode of transport based on clinical status at arrival, we found that among the 444 unstable patients, 270 arrived by ambulance, with 177 (65.5%) surviving, while 174 arrived by private vehicle, with 135 (77.5%) surviving (Table 3). Among the 1,214 stable patients, 134 arrived by ambulance, with 123 (91.7%) surviving, and 1,080 arrived by private vehicle, with 1,001 (92.6%) surviving over a period of 6 days (IQR: 4-10) (Table 4).

**Table 4:** Comparison of survival between patients arriving by ambulance versus other vehicles, stratified by triage status at arrival (N = 1,658*) ^*^ “Other vehicles” includes buses, two-wheelers, and four-wheelers.

Triage status	Ambulance		Other vehicles*		p-value	Chi-square value
	Number of children	Survival, n (%)	Number of children	Survival, n (%)	0.001	309.7944
Unstable (Levels 1 and 2) (N = 444)	270	177 (65.5)	174	135 (77.5)		
Stable (Levels 3-5) (N = 1,214)	134	123 (91.7)	1,080	1,001 (92.6)	-	-

## Discussion

This descriptive study aimed to assess the modes of transport and outcomes of children presenting to our pediatric emergency department. Over the study period, 1,658 children attended, with the largest proportion in the 60-144 month age group (N = 911, 54.9%), followed by the 1-12 month group (N = 747, 45%). The age distribution of our population aligns with findings from Bills et al. in northern India, who reported 39.1% of their cohort in the 2-12 month age group [6]. Among our study cohort, 1,016 (61.3%) were male, and 642 (38.7%) were female. The majority of referrals came from medical colleges (754 (45.4%)), followed by district hospitals (402 (24.2%)). These referral patterns are consistent with the study by Ezhumalai et al. in North India, which reported that 61.3% of their cohort were male, and most referrals originated from public sector hospitals, including medical colleges and district hospitals (85.4%) [4].

The leading reasons for presentation were respiratory complaints (477 (28.7%)), neurological conditions (271 (16.3%)), and renal issues (168 (10.1%)). Santhanam et al. from Chennai similarly found that respiratory illnesses (32%), fever (37%), and seizures (39%) were common reasons for admission [7]. The admission profile often reflects the local epidemiology and the availability of specialty care at the receiving hospital. Our facility, with a well-established pediatric ICU and nephrology unit, frequently receives referrals for intensive care and nephrology evaluations.

In terms of transport, the most common mode was by bus (654 (39.3%)), followed by ambulance (404 (24.3%)) and car (341 (20.8%)). Sankar et al. reported that auto-rickshaws (43.5%) were the most common mode of transport in Delhi [5]. In contrast, Ezhumalai et al. found that ambulances were used more frequently (85%) in their study population in North India [4]. The lower utilization of ambulances in our study may be due to limited accessibility, leading families to prefer locally available transport, such as buses. Additionally, 73.3% of the children in our study were stable upon arrival, possibly reducing the perceived need for ambulance services.

A notable finding was that 332 (20%) patients presented directly to our center without any prior external medical treatment. Despite being a referral center, one in five children did not receive prior care. Ezhumalai et al. reported that 73% of their patients were referred from other hospitals [4]. Our hospital’s location, on the border between two states, and the availability of round-the-clock pediatric emergency services likely contribute to this pattern of direct presentation. Additionally, our hospital does not require formal referral documentation or prior contact, which may further encourage direct arrivals.

Only 143 (10.8%) of referred patients had prior communication between their referring physicians and our hospital. Akande from Nigeria similarly reported that 92.9% of patients presented directly without a formal referral [8]. Sankar et al. from Delhi also noted a lack of prior communication before patient transfer [5]. The American Academy of Pediatrics emphasizes the importance of prior communication to optimize patient stabilization before transport [9]. In our study, 276 (16.6%) of children presented with hypothermia upon arrival, a preventable complication that could be mitigated by improved pre-transport communication and stabilization.

Critically ill patients are at risk of adverse events during transport to higher-level medical facilities. Hypothermia was more prevalent among those transported by private vehicles (224 (17.8%)) compared to ambulances (52 (12.8%)). Additionally, 60 (15%) of critically ill children transported by ambulance experienced desaturation (SpO₂ < 92%) at admission, compared to 105 (8.4%) in those transported by other vehicles.

Mortality rates were higher in children transported by ambulance (104 (25.7%)) compared to those transported by other vehicles (118 (9.4%)). This disparity may be due to the fact that more unstable patients were transported by ambulance. A stratified analysis based on triage status revealed that mortality was higher among unstable children (triage Levels 1 and 2) who arrived by ambulance (90 (34.5%)) compared to those transported by other vehicles (39 (22.5%)) (Table 4). Several factors could explain this observation. It is possible that children arriving by other vehicles were more stable at the time of referral, and their condition deteriorated during transport. Alternatively, the triage system may not fully capture the severity of illness, leading to underestimation of the clinical status in children transported by ambulance. A more comprehensive scoring system could provide deeper insights. Bills et al. found that patients with both respiratory distress and SpO₂ < 95% had nearly 10 times the odds of mortality compared to others [6]. Prabhudesai et al. in Chennai reported that 12.6% of children were transported by ambulance and 87.4% by private vehicles, with an overall mortality rate of 17.1% [10].

This study has limitations, including being single-centered and conducted over only eight months, which may affect its generalizability. Variations in disease epidemiology and healthcare infrastructure across regions and seasons also impact the applicability of our findings. Nevertheless, this study adds to the limited literature on pediatric prehospital transport practices in our country.

## Conclusions

Our study highlights the absence of a formal referral and transport system for patients referred to our tertiary care center. Despite this, certain outcomes, such as mortality, appear to be relatively unaffected by the mode of transport. However, further operational research is needed to evaluate the impact of structured referral communication and transport service on patient outcomes, particularly in the emergency unit and PICU. Such research could justify public health expenditure on these services in resource-limited settings like India.
